# UPLC-QTOF-MS metabolomics analysis revealed the contributions of metabolites to the pathogenesis of *Rhizoctonia solani* strain AG-1-IA

**DOI:** 10.1371/journal.pone.0192486

**Published:** 2018-02-06

**Authors:** Wenjin Hu, Xinli Pan, Fengfeng Li, Wubei Dong

**Affiliations:** 1 Department of Plant Pathology, College of Plant Science and Technology and the Key Lab of Crop Disease Monitoring & Safety Control in Hubei Province, Huazhong Agricultural University, Wuhan, Hubei Province, China; 2 Department of Biochemical and Chemical Engineering, Technische Universität Dortmund, Dortmund, Germany; Fujian Agriculture and Forestry University, CHINA

## Abstract

To explore the pathogenesis of *Rhizoctonia solani* and its phytotoxin phenylacetic acid (PAA) on maize leaves and sheaths, treated leaf and sheath tissues were analyzed and interpreted by ultra-performance liquid chromatography-mass spectrometry combined with chemometrics. The PAA treatment had similar effects to those of *R*. *solani* on maize leaves regarding the metabolism of traumatin, phytosphingosine, vitexin 2'' O-beta-D-glucoside, rutin and DIBOA-glucoside, which were up-regulated, while the synthesis of OPC-8:0 and 12-OPDA, precursors for the synthesis of jasmonic acid, a plant defense signaling molecule, was down-regulated under both treatments. However, there were also discrepancies in the influences exhibited by *R*. *solani* and PAA as the metabolic concentration of zeaxanthin diglucoside in the *R*. *solani* infected leaf group decreased. Conversely, in the PAA-treated leaf group, the synthesis of zeaxanthin diglucoside was enhanced. Moreover, although the synthesis of 12 metabolites were suppressed in both the *R*. *solani*- and PAA-treated leaf tissues, the inhibitory effect of *R*. *solani* was stronger than that of PAA. An increased expression of quercitrin and quercetin 3-O-glucoside was observed in maize sheaths treated by *R*. *solani*, while their concentrations were not changed significantly in the PAA-treated sheaths. Furthermore, a significant decrease in the concentration of L-Glutamate, which plays important roles in plant resistance to necrotrophic pathogens, only occurred in the *R*. *solani-*treated sheath tissues. The differentiated metabolite levels may be the partial reason of why maize sheaths were more susceptible to *R*. *solani* than leaves and may explain the underlying mechanisms of *R*. *solani* pathogenesis.

## Introduction

As one of the most important crops, maize has been cultivated widely around the world. In addition to providing food for people, maize is also used to produce bioenergy [1]. Maize production is reduced by various factors [2, 3]. Plant pathogens may cause severe yield losses. *Rhizoctonia solani*, a plant pathogen, is a necrotrophic fungus that has a wide range of plant hosts [4]. *R*. *solani* infection on plants primarily occurs on roots and lower stems. During the infection of *R*. *solani*, enzymes and small molecular toxins are released to damage host plants [5]. The known toxins isolated from *R*. *solani* are phenylacetic acid (PAA) and its derivatives [6]. However, the roles of PAA and its derivatives in the pathogenesis of *R*. *solani* on maize are not yet understood. Another secreted toxin was found to be a polysaccharide with a currently unknown structure [7]. There are a few reports about the interactions between maize and *R*. *solani*. When maize was infected by *R*. *solani*, modulations of gene expression involved in transcription, regulation, signal transduction, cellular transport, protein processing, metabolism, and defense were discussed [8, 9]. A novel PCR removal approach developed by our group was applied to isolate regulated genes in the maize leaf after the *R*. *solani* infection [10]. No research has been reported investigating the metabolomics of maize infected by *R*. *solani*.

Metabolomics is becoming a popular tool for studies in crops and pathogens. In maize, metabolic profiling-related studies have mostly been on environmental stresses and genetic modifications. Genetically modified and non-modified maize plants were compared and characterized by NMR metabolic profiles [11]. Metabolic variations related to osmolytes and branched amino acids were studied in the genetically modified maize [12]. Functional evaluation of the gene *hda101* was conducted in mutant maize plants by metabolic profiling, which confirmed its role in cell cycle control [13]. Accumulations of phenylalanine and tyrosine were found in maize mutants that were deficient for two glutamine synthetase isoenzymes using an NMR-based profiling technique [14]. When maize was treated with salt and profiled with NMR, the impact of high salinity on shoots was stronger than on roots [15]. Silk extracts from seven maize landraces were characterized based on NMR metabolic profiles in southern Brazil [16]. Impacts of genetics and the environment on the metabolic composition of maize grain were also evaluated [17, 18]. By metabolic profiling of six different maize hybrids in water deficiency together with data generated from morphophysiological measurements, several metabolites were shown to be correlated to drought and certain physiological traits [19]. Metabolic profiling was also conducted to analyze the genetic diversity of maize kernels [20]. Metabolite profiling and metabolic fingerprinting of the developmental stages and chemotaxonomy of *R*. *solani* species were also reported [21, 22].

To explore the effect of root treatments of *R*. *solani* and phenylacetic acid on leaf and sheath metabolism in maize, the ultra-performance liquid chromatography quadrupole time-of-flight mass spectrometry (UPLC-QTOF-MS) technique was applied to evaluate the metabolic alteration of the *Z*. *mays* inbred line B73 after treatment. The sheath and leaf samples from above ground were collected and analyzed using the UPLC-QTOF-MS technique. The structural information of metabolites was deduced from online database analysis using the MetFrag annotation method. The data were mainly analyzed using principal component analysis (PCA) and partial least square discriminant analysis (PLS-DA) as well as univariate Student’s t-test to compare variances among groups and metabolites. The changes in metabolites caused by *R*. *solani* or PAA were explored. Our aim is to explore the role of PAA as a phytotoxin in the pathogenesis of *R*. *solani* and to try to explain why maize sheaths are more susceptible to *R*. *solani* infection than leaves.

## Materials and methods

### Biological materials

The maize inbred line B73 and *Rhizoctonia solani* AG-1-IA were used in this study. After treatment with 7% hypochlorite solution for 30 min and washing with sterilized water, the seeds were sowed in pots with autoclaved moist composite soil. Seed germination and cultivation were performed in growth chambers with the following cycle conditions: 28/20°C (day/night) and 16/8 h (light/darkness). The *R*. *solani* AG-1-IA isolate was maintained on potato dextrose agar (PDA: 200 g/l, peeled potatoes; 20 g/l, dextrose; 15 g/l agar powder). Agar plugs from starter cultures were placed on PDA in Petri dishes (9 cm in diameters) and grown at 28°C in the dark. Based on the results of previous research, the concentration of PAA used for this study was set as 7.34 mM [23]. The PAA solution was prepared by dissolving 0.1 g of PAA (chemically pure) in 100 ml of distilled water and was filtrated through 0.22 μm filters. To test whether *R*. *solani* could infect maize tissues and PAA could cause the typical symptoms, a PDA plug (9 mm in diameters) with *R*. *solani* colonies and one filter paper (same size as the PDA plug) containing a PAA solution (7.34 mM, 200 μL) were placed on the surface of the maize sheaths, while one filter paper (9 mm in diameters) containing distilled water (200 μL) was used as a blank control. The results were observed after 48 hours.

### Sample collection of treated leaf and sheath tissues

To obtain metabolic responses in leaf and sheath tissues after maize plants were treated by *R*. *solani* and its phytotoxin PAA, the leaf and sheath tissues of the treated maize were analyzed. For each plant in the *R*. *solani-*treated group, three agar plugs (1 cm in diameter) cut from the growing edges of *R*. *solani* colonies with 1 ml distilled water were set close to the root, which was 3 cm under the soil. In the PAA-treated group, three PDA plugs and 1 ml of PAA solution were set close to the root of each plant. In the control group, three PDA plugs with 1 ml distilled water were placed close to the root of each plant. Inoculated plants were watered on the surface of the leaf and sheath once per 12 h to maintain a proper humidity. All of the maize plants were grown under the same conditions. Per our experience, it takes *R*. *solani* 24–48 hours to invade maize tissues successfully. Seventy-two hours, on one hand, should be sufficient for *R*. *solani* infection; on the other hand, the time point ensured that the maize tissue samples were not contaminated by metabolites from *R*. *solani* during the interactions. Then, the sheath samples were harvested 5 cm above the surface of the soil, and the leaf samples were harvested from the leaf closest to the ground where the sampling positions were 1 cm away from the sheath node. All of the samples were processed and inactivated immediately in liquid nitrogen. The location of the *R*. *solani* infection and the posterior sampling procedure on maize were illustrated in S1 Fig. Six groups were collected: three groups of maize leaves and three groups of maize sheaths. Each sample group consisted of five replicates.

### Metabolite extraction

The extraction of maize tissues followed the reported protocol with minor modifications [24]. Generally, the inactivated samples were ground into a fine powder using liquid nitrogen and were lyophilized in a frozen dryer for 48 hours. Fifty milligrams of each replicate and 1.5 ml of ice-cold methanol (UPLC graded, 0.1% HPLC graded formic acid) were added into a 2-ml tube. The samples were vortexed for 30 s and sonicated for 20 min in a water bath at 20°C. After centrifugation for 20 min at 14,000 *g* at room temperature, 1 ml of the upper suspensions was transferred into another 2-ml Eppendorf tube and was centrifuged for a second time at 4°C and 20,000 *g*. Suspensions of 0.8 ml were filtered into the auto samplers using 0.22 μm filters. Each sample was injected once for analysis. One sample was randomly selected from each group, and 0.1 ml from this sample was transferred to form a quality control (QC) sample. The QC sample was injected three times before the injection of maize leaf and sheath samples.

### UPLC-QTOF-MS analysis and data output

The Waters Ultra Performance Liquid Chromatography system (XEVO-G2XSQTOF#YEA677) was equipped with an ACQUITY C_18_ column (10 cm × 2.1 mm, particle size 1.7 μm, Waters, USA). The injection volume for each sample was 1 μL. The column was eluted using the following binary gradient solutions: A (deionized water with 0.1% formic acid) and B (acetonitrile, UPLC graded) with a flow rate of 0.4 ml/min: 99:1 at min 1, 80:20 at min 2, 60:40 at min 5, 45:55 at min 6, 20:80 at min 13, 5:95 at min 15 and 99:1 at min 20. The mass spectrometry was performed on the Waters QTOF MS equipped with an electrospray ionization source. The scan range was set from 50 Da to 1200 Da with a scan rate of 1 Hz. The mass detection was set both in positive and negative modes. The data were collected in the centroid mode. The low energy and high energy were set as 15 and 45 eV, respectively. The system parameters were referenced in our previous report and are listed concisely as follows: capillary voltage 1000 V, cone voltage 30 V, desolvation gas flow 800 l/h, desolvation temperature 400°C, cone gas flow 50 l/h, and source temperature 100°C [25]. The lock spray was set at 20 s to ensure accuracy and reproducibility, and leucine–enkephalin was used as the lock mass at a concentration of 0.8 ng/μL with a flow rate of 10 μL/min.

### Data analysis

The original data from the UPLC-QTOF-MS analysis were converted into four NetCDF datasets using the DataBridge unit of MassLynx (4.1). The first two CDF files were used for data analysis and metabolite annotation. The R package XCMS was used for the peak picking process with the following parameters: method “CentWave”, ppm 30, snthresh 10, peakwidth from 5 to 20. The parameters for peak grouping were set as follows: bw = 2, minfrac = 0.5, and mzwid = 0.015 [26]. The peak annotation was performed using the CAMERA package with default parameters [27]. The ion modes during the peak-picking and annotation procedures were consistent with those in the corresponding UPLC-QTOF-MS test.

After each variable was scaled in the column by the standard scaling method and normalized in the row, PCA was used for preliminary analysis, and PLS-DA combined with Student’s t-test was used for variable selection. The statistics were performed using the R environment (http://cran.r-project.org/) with the “ropls” package [28]. For each PLS-DA analysis, a predictive model was established, where the value R2X and R2Y was used to explain the variation of the model and the value Q2Y was used to estimate the ability of prediction. Each PLS-DA model was validated by performing the 7-fold validation-test and the Root Mean Square Error of Estimation (RMSEE) value. The 7 groups during validation were generated by the consecutive method. Further, the permutation test was used to evaluate whether each PLS-DA model was over-fitted or not (permutation times equal to 100). After the PLS-DA analysis, the variables that importantly contributed to the discrimination of groups were selected using the threshold VIP > 3 in each PLS-DA model. The significance of each variable was further verified using Student’s t-test with the threshold p-value < 0.01. The parent ions derived from the finally selected variables were used for the identification of structural information. The m/z value of each parent ion mass was used to retrieve metabolites using the online databases Metlin, KEGG and Lipidmaps with the parameter ppm less than 30. To obtain the best result among candidate compounds for each parent ion, every compound was first fragmented, and the fragmental information was aligned with the fragment peaks corresponding to the parent ion. The compound was then scored using the m/z values and signal intensities of matched peaks simultaneously, and the metabolite with the highest score was selected as the best solution [29].

## Results

### Exploratory analysis using the PCA method

**Fig 1:**
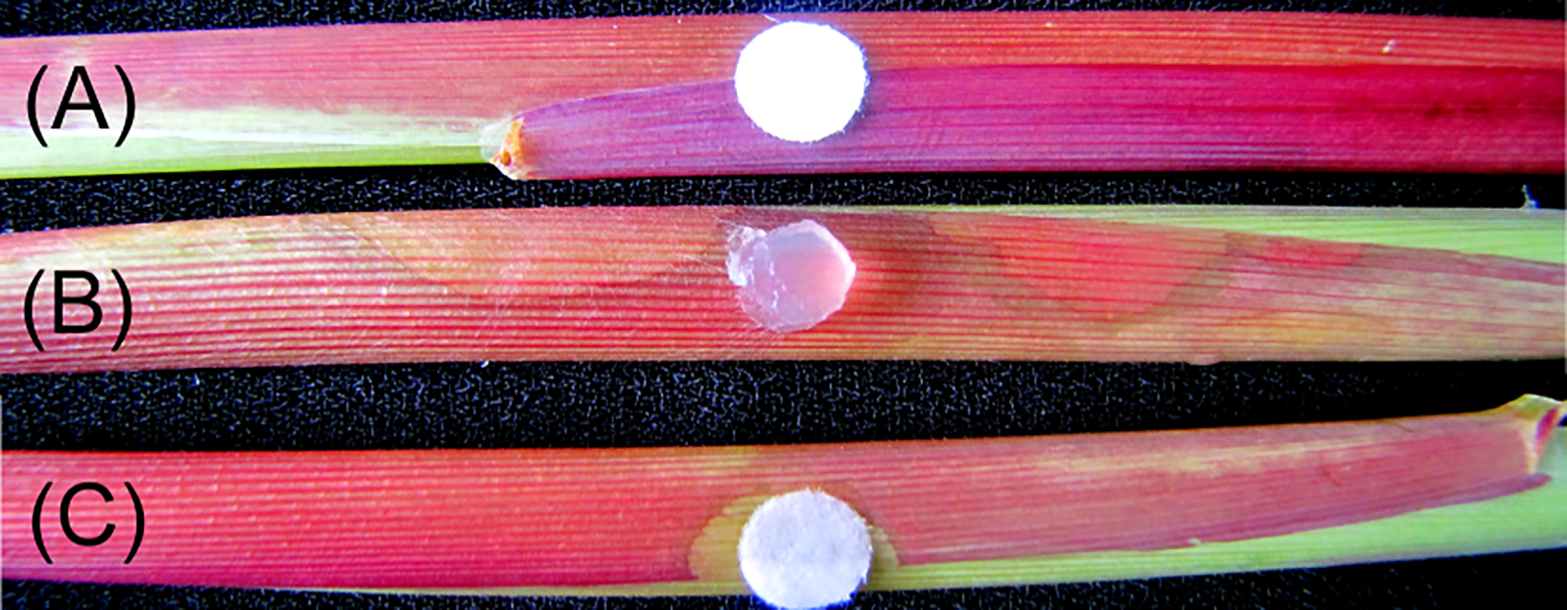
Symptoms induced in the maize sheaths inoculated by *Rhizoctonia solani* and phenylacetic acid. (A), filter paper (0.9 cm in diameters) with sterilized water. (B), a PDA plug (0.9 cm in diameters) cut from the growing edge of *R*. *solani* AG-1IA colonies. (C), filter paper (0.9 cm in diameters) with phenylacetic acid solution (7.34 mM, 200 μL, filtered by a 0.22 μm filter).

Symptoms on maize sheaths due to *R*. *solani* and PAA treatments are presented in Fig 1. Compared to the blank control, the *R*. *solani* and PAA treatments both caused severe disease symptoms in the sheath tissues. Compared with *R*. *solani*, PAA resulted in faded pigments in sheath tissues, and the cell necrosis caused by PAA in sheaths was similar to those that were caused by *R*. *solani*. The PAA was an effective phytotoxin in the process of *R*. *solani* infection.

**Fig 2:**
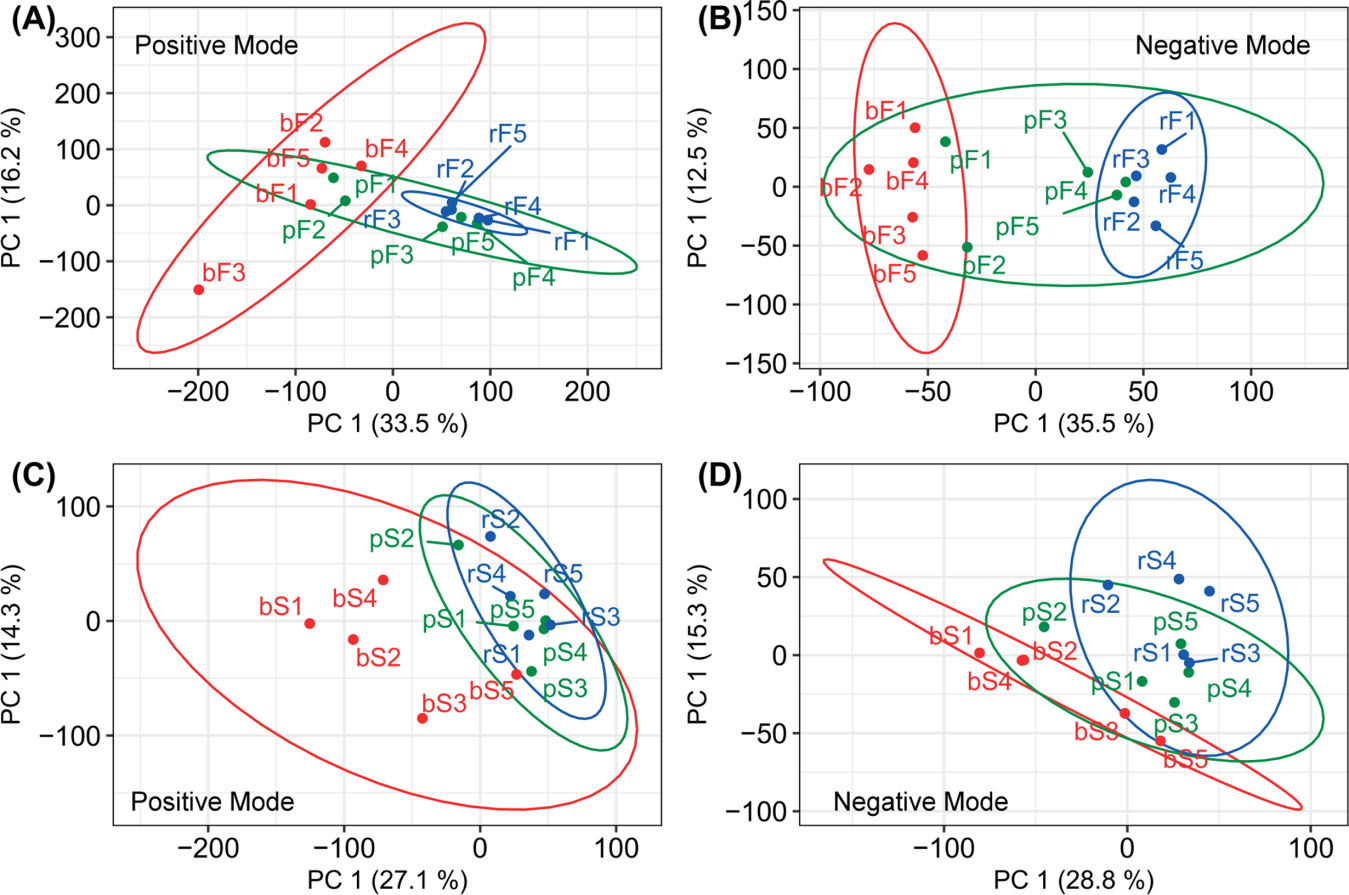
Score plots for the principal component analysis. (A) and (B), the score plots based on the first two components derived from the PCA analysis of the leaf tissues analyzed in positive and negative ion modes, respectively. (C) and (D), the score plots based on the first two components derived from the PCA analysis of the sheath tissues analyzed in positive and negative ion modes, respectively. In the PCA results (A), (B), (C) and (D), the first two components explained 49.7%, 48.0%, 41.4% and 44.1% of the total variances, respectively. Samples bF 1–5, pF 1–5 and rF 1–5 were collected from the control, the phenylacetic acid and *R*. *solani* treated leaf tissues, respectively. Samples bS 1–5, pS 1–5 and rS 1–5 were collected from the control, phenylacetic acid and *R*. *solani* treated sheath tissues, respectively.

The leaf and sheath samples for UPLC-QTOF-MS spectra analyses were collected from the *Zea mays* inbred line B73. Six groups were collected, and there were five replicates for each group. The infection and sampling procedure was presented in S1 Fig. The data generated from the UPLC-QTOF-MS tests for maize leaves and sheaths were presented as S1–S4 Tables. The PCA was applied for the preliminary analysis. Due to the inherent difference in the metabolism of the maize blade and sheath tissues, they were analyzed separately. For the leaf samples, the PCA analyses for the positive and negative ion modes were constructed, and their score plots based on the first two principal components were presented as Fig 2(A) and 2(B). The first two components explained 49.7% and 48.0% of the total variances in the positive and negative ion mode, respectively. The control samples separated clearly from the *R*. *solani*- and PAA-treated samples along the PC1 direction. However, the *R*. *solani*- and the PAA-treated samples were not different. The results suggested that the *R*. *solani* and PAA treatments both induced changes in maize leaf metabolism and that the changes in the maize leaf induced by the two treatments overlapped. For the sheath samples, the PCA analysis was performed separately for the positive and negative ion modes, and their individual score plots were constructed based on the first two components and were presented in Fig 2(C) and 2(D). The first two components explained 41.4% and 44.1% of the total variances for the positive and negative ion modes, respectively. Per the score plot for the positive ion mode, the control samples separated clearly from the *R*. *solani*- and PAA-treated samples along the PC1 direction, while the separation of two treatments was not clear. The results showed that there were similarities and differences in metabolic changes caused by *R*. *solani* and PAA in the sheath samples. The results in the negative ion mode were not ideal because the separation among the three groups was not clear. Due to the complexity in the metabolic composition of maize tissues, the results derived from PCA analysis were still obscure.

### Supervised analysis of samples and metabolite identification

**Fig 3:**
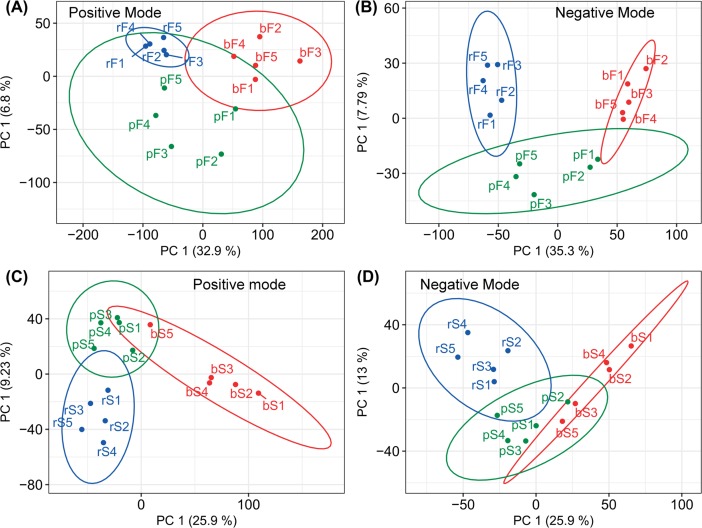
Score plots for the PLS-DA results. The score plots based on the first two components derived from the PLS-DA results for the leaf tissues analyzed in positive and negative ion modes were presented as (A) and (B), respectively. The score plots based on the first two components derived from the PLS-DA analysis results for the sheath tissues analyzed in positive and negative ion modes were presented as (C) and (D), respectively. The first two components in the PLS-DA models (A), (B), (C) and (D) explained 50.6%, 43.1%, 35.1% and 38.9% of the total variances, respectively. Samples bF 1–5, pF 1–5 and rF 1–5 were from the control, the phenylacetic acid and *R*. *solani* treated leaf tissues, respectively. Samples bS 1–5, pS 1–5 and rS 1–5 were from the control, the phenylacetic acid and *R*. *solani* treated sheath tissues, respectively.

A PLS-DA model was built to investigate the variance among the three groups of two maize tissues, separately. The score plots derived from the PLS-DA models built for the leaf tissues tested in positive and negative ion modes are presented in Fig 3(A) and 3(B). Their corresponding permutation test plots are presented in S2(A) and S2(B) Fig. For the positive ion mode of the leaf samples, the PLS-DA model was built based on the first three latent components, which explained 50.6% (R2X) of the total variance. In this PLS-DA model, R2Y, Q2Y and RMSEE were 0.884, 0.463, and 0.187, respectively. For the leaf samples tested in the negative ion mode, the PLS-DA model was built on the first two latent components, which explained 43.1% (R2X) of the total variance. Its R2Y, Q2Y and RMSEE were 0.854, 0.486, and 0.202, respectively. The score plots derived from the PLS-DA models built for the sheath tissues tested in positive and negative ion modes are presented in Fig 3(C) and 3(D). Their corresponding permutation test plots are presented in S2(C) and S2(D) Fig. For the sheath tissues tested in the positive ion mode, the PLS-DA model was built on the first two components, which explained 35.1% (R2X) of the total variance. The R2Y, Q2Y and RMSEE for this PLS-DA model were 0.795, 0.319 and 0.238, respectively. For the sheath tissues tested in the negative ion mode, the first two latent components were used and explained 38.9% (R2X) of the total variance. The R2Y, Q2Y and RMSEE in the PLS-DA model were 0.725, 0.289 and 0.276, respectively. The results suggested that for the leaf and sheath samples tested in both ion modes, a separation was clearly observed between the two treated and control samples. The results suggested that changes in metabolism were induced by both treatments. After multivariate and univariate analyses were performed, the selected parent ions were retrieved using online databases to obtain the corresponding candidate compounds. For each parent ion, the retrieved compounds were analyzed in three steps to select the optimal one, including the fragmentation of compounds, the alignment of the fragmented peaks, and the scoring based on the matched fragmented peaks. In addition to L-Glutamate and DIBOA-glucoside, a range of fatty acids, phospholipids, flavonoids, carotenoids, and alkaloids have been identified as having significant changes in their concentrations from extracts of maize tissues. The identified metabolites are listed in Table 1.

**Table 1:** Identification of metabolites with significant changes in expression.

Parent mass	Mass	Adduct	Mass error (ppm)	Name	Tissues
175.1120	192.1150	[M+H-H2O]	5.2	thymyl acetate	Leaf
213.1485	212.1412	[M+H]+	3.8	traumatin	Leaf
275.2006	274.2005	[M+H]+	20.1	(2R,3R)-3-Methylornithinyl-N6-lysine	Leaf
320.0946	281.1263	[M+K]+	20.3	harzianopyridone	Leaf
275.2007	292.2038	[M+H-H2O]	4.1	12-OPDA	Leaf
295.2265	294.2195	[M+H]+	1.7	OPC-8:0	Leaf
298.2740	297.2668	[M+H]+	4.0	cassine	Leaf
289.1801	306.1831	[M+H-H2O]	2.9	capsiate	Leaf
333.2039	310.2144	[M+Na]+	1.3	13(S)-HPOT	Leaf
335.2190	312.2301	[M+Na]+	0.3	13(S)-HPODE	Leaf
318.3002	317.2930	[M+H]+	3.2	phytosphingosine	Leaf
342.0818	343.0903	[M-H]-	6.7	DIBOA-glucoside	Leaf
369.0016	370.0066	[M-H]-	6.5	D-glycero-beta-D-manno-Heptose 1,7-bisphosphate	Leaf
403.2479	402.2406	[M+H]+	1.0	dehydrocholic acid	Leaf
489.3571	488.3502	[M+H]+	4.5	asiatic acid	Leaf
625.4320	586.4750	[M+K]+	20.5	3',4'-Dihydrorhodovibrin	Leaf
593.1513	594.1585	[M-H]-	2.5	vitexin 2''-O-beta-D-glucoside	Leaf
609.1447	610.1534	[M-H]-	0.7	rutin	Leaf
649.4098	610.4386	[M+K]+	28.5	2-Ketospirilloxanthin	Leaf
745.4122	722.4440	[M+Na]+	27.7	prephytoene diphosphate	Leaf
761.5846	760.5856	[M+H]+	7.4	PC(16:0/18:1(9Z))	Leaf
797.5162	774.5282	[M+Na]+	8.0	MGDG(18:3(9Z,12Z,15Z)/18:3(9Z,12Z,15Z))	Leaf
891.5227	892.5337	[M-H]-	0.3	zeaxanthin diglucoside	Leaf
146.0444	147.0532	[M-H]-	1.4	L-Glutamate	Sheath
211.0219	188.0321	[M+Na]+	11.2	cis-Homoaconitate	Sheath
376.2596	375.2410	[M+H]+	29.3	icaceine	Sheath
381.0895	380.0896	[M+H]+	30.5	diphyllin	Sheath
447.0922	448.1006	[M-H]-	5.8	quercitrin	Sheath
465.1034	464.0955	[M+H]+	1.1	quercetin 3-O-glucoside	Sheath
551.5017	550.4961	[M+H]+	0.2	DG(P-14:0/18:1(9Z))	Sheath
599.4073	576.4179	[M+Na]+	12.0	3-Hexaprenyl-4-hydroxy-5-methoxybenzoate	Sheath
601.4235	600.4179	[M+H]+	0.2	capsorubin	Sheath
609.1459	610.1534	[M-H]-	0.7	rutin	Sheath
617.1476	594.1585	[M+Na]+	0.8	vitexin 2''-O-beta-D-glucoside	Sheath

### Metabolites significantly changed in treated leaf tissues

**Fig 4:**
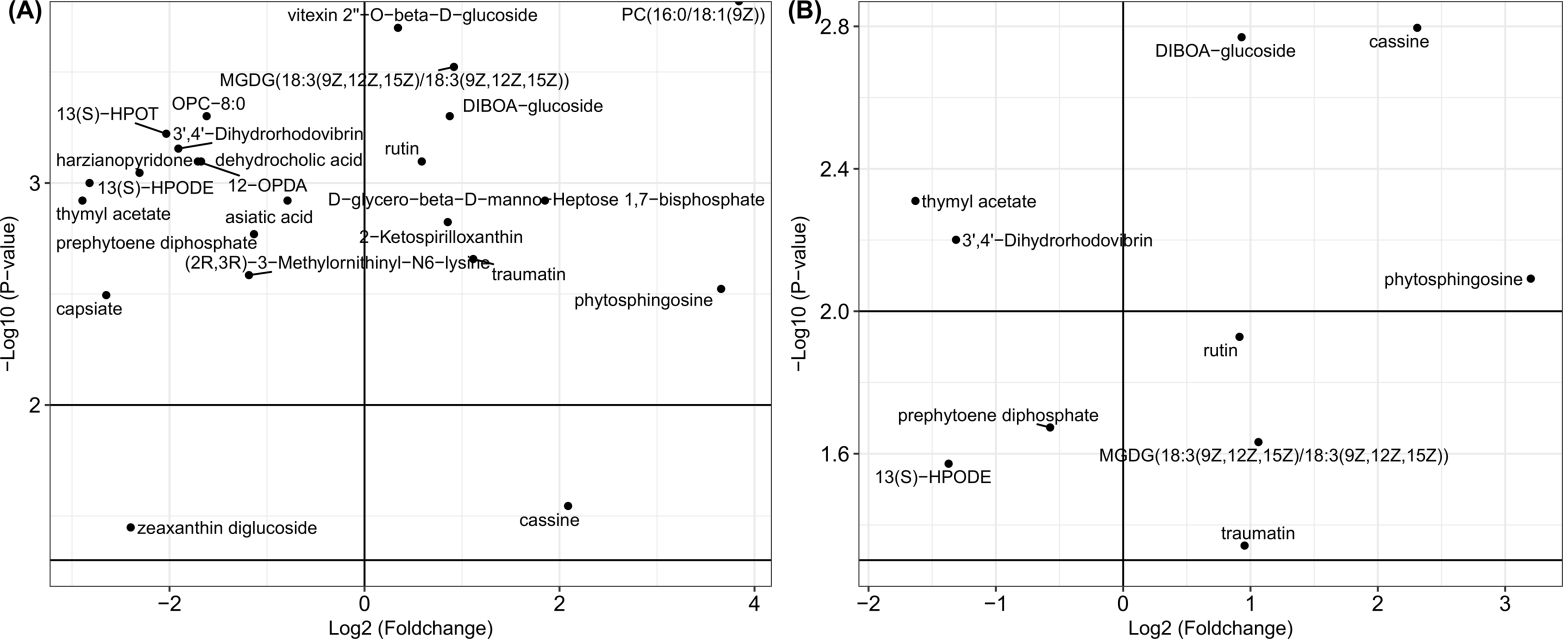
Scatter plots of the metabolites significantly changed in leaf tissues. (A), the scatter plot of metabolites that significantly changed in the *R*. *solani* infected group. (B), the scatter plot of the metabolites that significantly changed in the phenylacetic acid treated group. The fold change value for each metabolite was Log2 transformed, and the corresponding p-value was -Log10 transformed.

The mass abundances for corresponding metabolites in each sample and the fold change value and p-value for each metabolite among the three groups are presented in S5 and S6 Tables. Metabolites with expression fold changes (Log2 transformed) in leaves treated by *R*. *solani* and PAA are presented in Fig 4(A) and 4(B), separately. The metabolic levels of 10 metabolites were elevated in the *R*. *solani*- and PAA-treated groups. The expressions of traumatin, MGDG(18:3(9Z,12Z,15Z)/18:3(9Z,12Z,15Z)), phytosphingosine, rutin, cassine and DIBOA-glucoside were all significantly (p-value < 0.01) elevated (fold change value > 1) in both leaf treatment groups. Additionally, the expression levels of D-glycero-beta-D-manno-heptose 1,7-bisphosphate, vitexin 2"-O-beta-D-glucoside, PC(16:0/18:1(9Z)) and 2-ketospirilloxanthin in the *R*. *solani*-treated leaves were 3.61, 1.27, 14.32 and 1.81 times higher than those in the control group, respectively (p-value < 0.01). The expression of zeaxanthin diglucoside was increased by 1.27-fold in the PAA-treated group, but its expression was inhibited by 0.19-fold in the *R*. *solani* group (p-value < 0.05).

There were 12 metabolites with decreased expression in the *R*. *solani*- and PAA-treated leaf tissues compared to the control. Among them, the expressions of metabolites in both treatment groups, such as prephytoene diphosphate, 13(S)-HPODE, thymyl acetate and 3',4'-dihydrorhodovibrin, were significantly (p-value < 0.05) inhibited. The fold change values of the expressions of 12-OPDA, OPC-8:0, and 13(S)-HPOT were decreased by 0.31, 0.33, and 0.24, respectively, in the *R*. *solani*-treated leaves (p-value < 0.001). The inhibition of the metabolic expressions of 12 metabolites in both treatment groups is presented in S3 Fig, in which the results suggested that the inhibition by *R*. *solani* was stronger than that of PAA. Compared with PAA, *R*. *solani* is suggested to have a stronger inhibitory effect on the synthesis of these metabolites.

### Metabolites significantly changed in treated maize sheath tissues

**Fig 5:**
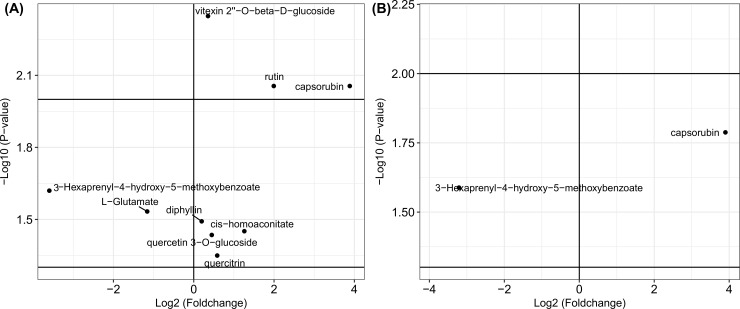
Scatter plots of the metabolites significantly changed in sheath tissues. (A), the scatter plot of the metabolites that significantly changed in the *R*. *solani* infected group. (B), the scatter plot of the metabolites that significantly changed in the phenylacetic acid treated group. The fold change value for each metabolite was Log2 transformed, and the corresponding p-value was -Log10 transformed.

The metabolites with significant changes in their synthesis among sheath groups treated by *R*. *solani* and PAA are presented in Fig 5(A) and 5(B), separately. In both treated sheaths, the expression of capsorubin was increased, while the metabolic level of 3-Hexaprenyl-4-hydroxy-5-methoxybenzoate was inhibited. In the *R*. *solani*-treated group, the fold change values in the metabolic levels of quercitrin, cis-homoaconitate, quercetin 3-O-glucoside, vitexin 2"-O-beta-D-glucoside, and rutin were 1.50, 2.40, 1.37, 1.28, and 3.98 times higher than that of corresponding metabolites in the control group (p-value < 0.05), respectively. Additionally, in the *R*. *solani-*treated group, the expression of L-Glutamate was inhibited by 0.45-fold (p-value < 0.05).

## Discussion

During the infection process, degradation enzymes and toxins play major roles in the pathogenesis of necrotrophic pathogens. Plants can prevent the further infection of pathogens by strengthening the cell walls, initiating oxidative cross-linking reactions to produce lignin and antimicrobial proteins, or forming physical barriers that are resistant to pathogen infection. Based on these pathogenesis strategies, we speculated that metabolic profiling should be an efficient method to explore the mechanisms of interactions between plants and necrotrophic pathogens. *R*. *solani* is a typical necrotrophic pathogen that has a wide host range. Our results demonstrated that metabolic profiling is an efficient strategy to explore the pathogenesis of necrotrophic pathogens and the mechanisms of susceptibility or resistance of maize. Our results revealed that *R*. *solani* could up-regulate a range of metabolites. Some key metabolites related to different pathways were involved in maize resistance to *R*. *solani* infection. Metabolites that should be associated with the successful infection of *R*. *solani* were also revealed.

### Fatty acids and lipids related to signal transduction

Expressions of detected fatty acids varied. In leaf tissues, the metabolism of traumatin was suggested to respond to both *R*. *solani* and PAA treatments as the concentrations were elevated in both treatment groups. Traumatin is an oleic acid and is involved in the metabolism of alpha-linolenic acid. As a plant hormone, it responds to plant wounds [30]. It cooperates with other plant hormones to stimulate growth, adapt to stresses, and regulate metabolic pathways [31]. Its application enhances the activity of various antioxidant enzymes, including sodium dismutase, catalase, ascorbate peroxidase, NADH peroxidase, and glutathione reductase [32]. The metabolisms of 12-OPDA, OPC-8:0 and 13(S)-HPOT, which are alpha-linolenic acids, were down-regulated in the *R*. *solani*- and PAA-treated leaf tissues. OPC-8:0 and 12-OPDA are precursors used for the synthesis of jasmonic acid (JA). JA is recognized as an important signaling molecule involved in various pathways that can respond to different biotic or abiotic stresses. It is speculated that oxidation regulation of unsaturated fatty acids can be used for long-distance transport in wounded plants [33]. The speculation is consistent with similar studies since the expression levels of mRNA that are involved in several JA synthesis pathways in long distance transport were not significantly increased in injured tomato plants [34]. The enhanced expression of long chain fatty acids in *Arabidopsis thaliana* is closely related to promoting the antibacterial ability of *A*. *thaliana* [35]. The metabolism of fatty acids suggested that traumatin-dependent pathways were increased to respond to *R*. *solani* and PAA treatments while the JA-mediated defense pathways were not significantly enhanced.

There were also differences in the expression levels of phospholipids. The concentrations of MGDG (18:3(9Z,12Z,15Z)/18:3(9Z,12Z,15Z)) and PC (16:0/18:1(9Z)), both of which are phospholipids, were elevated in both treated leaf tissues. The metabolic level of DG (P-14:0/18:1(9Z)) was decreased in both treated sheath tissues. Phospholipids often act as signaling molecules during the interaction between plants and microorganisms. The infection of plant tissues induces changes in the synthesis, modification or redistribution of lipid metabolism. There are three ways to regulate the interactions between plants and pathogens by lipids, including producing oxylipin or JA in the lipoxygenase pathway, remodeling the membrane lipid composition and defense signaling in the unsaturated fatty acid pathways, and synthesizing very long chain fatty acids [36]. The expressions of phospholipids were elevated in rice, tomato, potato and *A*. *thaliana* and other plants after pathogen infection or treatment by induction factors [37–40]. Phytosphingosine, a type of sphingolipid, was increased in both treated leaf groups. Sphingolipids play important roles in membrane structures and signal transduction in eukaryotic cells. Plant sphingolipids have important roles in the processes of programmed cell death (PCD), HR reaction, ABA-dependent defense cell closure, pathogen-plant interactions, and signal transduction under abiotic stresses [41]. Sphingolipids may identify interactions between plants and pathogens and induce plants to initiate a defense. In a double knockout mutant of *A*. *thaliana*, a decrease in resistance to diseases and the significant reduction in sphingolipid levels were observed [42]. When the *R*. *solani* infection or PAA treatment occurs at the root, enhancing the cell wall to resist infection may be a preferred strategy for maize.

### Carotenoids and flavonoids resistant to infection

In leaf tissues, the metabolic levels of zeaxanthin diglucoside were increased in the PAA-treated group but decreased in the *R*. *solani*-infected group. Additionally, the expression of capsorubin was increased in both treated sheaths. The functional metabolism of carotenoids includes stabilization of membrane lipid bilayers, removal of free radicals caused by ROS, and protection against membrane lipid peroxidation [43–45]. Carotenoids can be used as signal molecules to control mycorrhizal colonization [46]. Some carotenoid lysates are root-specific and can accumulate well after the inoculation of mycorrhizal fungi [47]. The metabolic differences of canthaxanthin and zeaxanthin in the two treated maize leaves suggested that the applications of *R*. *solani* and PAA resulted in different metabolic responses and reflected differences in the infection patterns of *R*. *solani* and PAA.

In the leaf and sheath tissues under both treatments, for the metabolism of flavonoids, the concentrations of vitexin 2''-O-beta-D-glucoside and rutin were higher than in the corresponding blank control groups. Furthermore, the concentrations of quercitrin and quercetin 3-O-glucoside in the two treated sheath samples were also increased. Flavonoids are produced from shikimic acid in plant phenylpropanoid pathways, from by-products of lignin pathways, or from decomposition products of lignin and cell wall polymers. The functions of flavonoids include UV protection, antioxidation, pigments, plant auxin transport regulators, anti-pathogenic defense compounds and regulators in the symbiotic process [48]. Flavonoids can inhibit the formation of ROS by inhibiting related enzyme activities, chelating trace elements during free radical production, and removing reactive substances or regulating antioxidant defense [49]. Flavonoids can also inhibit different pathogens and pests, including bacteria, fungi and insects [50]. The flavonoids in seeds or roots can be used to protect against diseases or insects [51]. Rutin can inhibit inflammation and shows a strong antioxidation effect. Rutin has a specified antibacterial effect on bacteria, including *Xanthomonas campestris*, *Agrobacterium tumefaciens*, and *Xylella fastidiosa* [52, 53]. Additionally, rutin can be used to reduce the normal physiological functions of reactive oxygen species in microbes [54]. The application of 2 mM rutin can induce rice, tobacco, and *A*. *thaliana* resistances to *Xanthomonas oryzae* pv. *oryzae*, *Ralstonia solanacearum*, and *Pseudomonas syringae* pv. *tomato* strain DC3000, respectively. The functional metabolism of rutin is also presumably associated with the SA-dependent signaling pathway [55]. Quercetin, a widely distributed flavonoid, shows inhibitory activities in the conidial germination test of fungus *Neurospora crassa* [56].

### The enhanced synthesis of DIBOA-glucoside in leaves

DIBOA-glucoside, a benzoxazinoid, had elevated concentrations in both treated leaf groups. DIBOA-glc is a 1,4-benzoxazine-3-one (BX), which is an important secondary metabolite for defense in maize. The antimicrobial activity against phytopathogens of BXs can be induced by aphids, worms, pathogens or plant rhizosphere-promoting bacteria (PGPR) [57–60]. Among them, DIMBOA, which is synthesized from DIBOA, is the major BX in maize against herbivorous insects and pathogens [61]. When treated with elicitors from the pepper pathogen *Phytophthora capsici*, the contents of BXs (DIBOA and DIMBOA) and the expressions of defense-related genes in maize roots and shoots were increased [62].

### Glutamate metabolism in sheath tissues was inhibited by *R*. *solani* infection

The concentration of L-Glutamate was significantly decreased in the *R*. *solani-*treated sheath groups. Glutamate plays vital roles in the interactions between plants and necrotrophic pathogens with functions in nitrogen transport, cellular redox regulation, and energy reprogramming in the tricarboxylic acid cycle. When plants are infected by biotrophs or necrotrophs, the metabolic changes of glutamate are determined by the response strategies from plant hosts or the strategies adopted by pathogens. The host cell death during the necrotrophic pathogen infection may be due to the N deficiencies in the Gln synthetase (GS)/Gln-oxoglutarate aminotransferase (GOGAT) cycle, ROS generation, or TCA cycle [63]. The virulence strategies of necrotrophic pathogens include overconsumption of cytosolic glutamate, inhibition of the GS/GOGAT-supplying glycine decarboxylase (GDC) enzyme, overactivation of Glu dehydrogenase (GDH) used for facilitating host cell death, and reduction in the levels of glutathione (GSH) for inhibiting the host antioxidant capacity [64–66]. However, it is still unknown whether the inhibition in the concentration of L-Glutamate is due to the strategies of maize resistance or the strategies of *R*. *solani* infection. L-Glutamate metabolism was significantly inhibited only in the sheaths of *R*. *solani*-treated groups. This may be one of the reason of maize sheath being more susceptible to *R*. *solani* infection than the maize leaf. Additionally, our results once again illustrated the complexities of the pathogenesis of *R*. *solani* because the PAA did not significantly alter the glutamate concentration in sheaths. This point was also confirmed by the comparison between symptoms caused by *R*. *solani* and PAA on sheaths.

## Conclusion

Metabolomics is a promising tool to investigate the interactions between two maize tissues and the necrotrophic pathogen *R*. *solani* as well as its phytotoxin PAA. A number of metabolites with significant changes in their concentrations were observed in the leaf and sheath tissues when the biotic stresses from *R*. *solani* and abiotic stresses from PAA occurred on the roots. Our results revealed that PAA played an important role in the pathogenesis of *R*. *solani* in maize tissues as PAA elicited changes similar to *R*. *solani* on the metabolism of maize. In both treated leaf tissues, the synthesis of traumatin, MGDG(18:3(9Z,12Z,15Z)/18:3(9Z,12Z,15Z)), DIBOA-glucoside and PC(16:0/18:1(9Z)) were elevated. The concentrations of OPC-8:0 and 12-OPDA, which are precursors for the synthesis of jasmonic acid, were reduced in both treated leaf tissues. The increased concentration of phytosphingosine, a type of sphingolipid, was observed in two types of treated leaves. The concentrations of flavonoids such as vitexin 2''-O-beta-D-glucoside and rutin were up-regulated in the leaf and sheath tissues of both treated groups. However, our results also demonstrated variations in the influences of PAA and *R*. *solani* on maize metabolism. In maize leaves, there were 12 metabolites whose synthesis was inhibited in both treated groups, and *R*. *solani* showed a stronger inhibitory effect on the synthesis of these metabolites than PAA. Further, in the *R*. *solani* treated leaves, the expression of zeaxanthin diglucoside was decreased, while in the PAA treated leaves, its synthesis was increased. Meanwhile, the significantly increased expressions of quercitrin and quercetin 3-O-glucoside were only observed in the *R*. *solani-*treated sheaths. The metabolic responses for resistance in the two maize tissues varied after *R*. *solani* infection of the roots because elevated concentrations of quercitrin and quercetin 3-O-glucoside were observed in the sheaths, while the concentrations of DIBOA-glucoside were increased in the leaves. Furthermore, the concentration of L-Glutamate, which plays key roles in plant resistances to necrotrophic pathogens, was significantly suppressed only in the sheath tissues of the *R*. *solani* infected group.

## Supporting information

S1 Table The UPLC-QTOF-MS test in positive modes for maize leaves. (CSV)

S2 Table The UPLC-QTOF-MS test in negative modes for maize leaves. (CSV)

S3 Table The UPLC-QTOF-MS test in positive modes for maize sheaths. (CSV)

S4 Table The UPLC-QTOF-MS test in negative modes for maize sheaths. (CSV)

S5 Table The mass abundances of corresponding metabolites in each sample. (CSV)

S6 Table Fold changes and their significances for each metabolite among groups of leaf and sheath tissues. Gpb: Each value represented the ratio of the average concentration of a given metabolite in the phenylacetic acid treated group divided by the average concentration of the same metabolite in the control group. Grp and Grb: Each value represented the ratio of the average concentration of a given metabolite in the *Rhizoctonia solani* infected group divided by the average concentration of the same metabolite in the phenylacetic acid treated group and the control group, respectively. The leaf and sheath tissues were compared separately. ***: p-value < 0.001. **: p-value < 0.01. *: p-value < 0.05.
(DOCX)

S1 Fig Illustration of the inoculation of *Rhizoctonia solani* and phenylacetic acid and the sampling of sheath tissues. The inoculation of group A consisted of three PDA plugs with *R*. *solani* colonies and 1 ml distilled water. The inoculation of group B consisted of three PDA plugs and 1 ml phenylacetic acid solution. The inoculation of group C consisted of three PDA plugs and 1 ml distilled water. The leaf tissues that were close to the ground and 1 cm away from the sheath node were collected. The sheath tissues that were 5 cm away from the ground were sampled.
(TIF)

S2 Fig The plots derived from the permutation tests for each PLS-DA model. (A) and (B), the plots from the permutation test results of the PLS-DA models for the leaf tissues analyzed in positive and negative ion modes, respectively. (C) and (D), the plots from the permutation test results of the PLS-DA models for the sheath tissues analyzed in positive and negative ion modes, respectively. The number of each permutation test equals 100. The results suggested that all PLS-DA models were not over-fitted.
(TIF)

S3 Fig The bar plot used to express the inhibitory ratios in the expressions of 12 metabolites in the *Rhizoctonia solani* and phenylacetic acid treated leaf groups. Grb and Gpb: Each value represented the ratio of the average concentration of a given metabolite in the *R*. *solani* (Grb) or phenylacetic acid (Gpb) treated leaf group divided by the average concentration of the same metabolite in the control leaf group.
(TIF)
