# Supplementary material for: UPLC-QTOF-MS metabolomics analysis revealed the contributions of metabolites to the pathogenesis of Rhizoctonia solani strain AG-1-IA
Source: PLoS One. 2018 Feb 6;13(2):e0192486. doi: 10.1371/journal.pone.0192486 (PMC5800620; doi:10.1371/journal.pone.0192486)
Supplement: S6 Table — Gpb: Each value represented the ratio of the average concentration of a given metabolite in the phenylacetic acid treated group divided by the average concentration of the same metabolite in the control group. Grp and Grb: Each value represented the ratio of the average concentration of a given metabolite in the Rhizoctonia solani infected group divided by the average concentration of the same metabolite in the phenylacetic acid treated group and the control group, respectively. The leaf and sheath tissues were compared separately. ***: p-value < 0.001. **: p-value < 0.01. *: p-value < 0.05. (DOCX) [file pone.0192486.s006.docx]

| Name | Grb | Grp | Gpb | Tissue |
| --- | --- | --- | --- | --- |
| (2R,3R)-3-Methylornithinyl-N6-lysine | 0.4397** | 0.6914 | 0.636 | leaf |
| 12-OPDA | 0.3057*** | 0.5768 | 0.53 | leaf |
| 13(S)-HPODE | 0.1416** | 0.3664 | 0.3866* | leaf |
| 13(S)-HPOT | 0.2443*** | 0.4741 | 0.5154 | leaf |
| 2-Ketospirilloxanthin | 1.807** | 1.24 | 1.4572 | leaf |
| 3',4'-Dihydrorhodovibrin | 0.2662*** | 0.6614 | 0.4025** | leaf |
| asiatic acid | 0.5783** | 0.835 | 0.6926 | leaf |
| capsiate | 0.1594** | 0.326 | 0.4889 | leaf |
| cassine | 4.2506* | 0.8575 | 4.9571** | leaf |
| D-glycero-beta-D-manno-Heptose 1,7-bisphosphate | 3.6067** | 2.5855** | 1.395 | leaf |
| dehydrocholic acid | 0.3129*** | 0.3366 | 0.9297 | leaf |
| DIBOA-glucoside | 1.833*** | 0.9621 | 1.9053** | leaf |
| harzianopyridone | 0.202*** | 0.4367 | 0.4626 | leaf |
| MGDG(18:3(9Z,12Z,15Z)/18:3(9Z,12Z,15Z)) | 1.888*** | 0.9041 | 2.0883* | leaf |
| OPC-8:0 | 0.3255*** | 0.5874 | 0.5541 | leaf |
| PC(16:0/18:1(9Z)) | 14.3243*** | 7.3145*** | 1.9584 | leaf |
| phytosphingosine | 12.6156** | 1.3716 | 9.1976** | leaf |
| prephytoene diphosphate | 0.4557** | 0.6782 | 0.6719* | leaf |
| rutin | 1.5024*** | 0.7975 | 1.8839* | leaf |
| thymyl acetate | 0.1346** | 0.4172 | 0.3227** | leaf |
| traumatin | 2.166** | 1.1185 | 1.9365* | leaf |
| vitexin 2''-O-beta-D-glucoside | 1.2685*** | 1.0368 | 1.2235 | leaf |
| zeaxanthin diglucoside | 0.1897* | 0.1494*** | 1.27 | leaf |
| 3-Hexaprenyl-4-hydroxy-5-methoxybenzoate | 0.0874* | 0.8085 | 0.1081* | sheath |
| capsorubin | 14.7784** | 0.9907 | 14.917* | sheath |
| cis-Homoaconitate | 2.3955* | 1.9487 | 1.2293 | sheath |
| DG(P-14:0/18:1(9Z)) | 0.3098 | 0.7072** | 0.438 | sheath |
| diphyllin | 1.1471* | 1.0876 | 1.0548 | sheath |
| icaceine | 0.6331 | 0.4616* | 1.3717 | sheath |
| L-Glutamate | 0.4481* | 0.619 | 0.7239 | sheath |
| quercetin 3-O-glucoside | 1.3652* | 1.2862 | 1.0614 | sheath |
| quercitrin | 1.5001* | 1.3083 | 1.1466 | sheath |
| rutin | 3.9804** | 1.3394 | 2.9717 | sheath |
| vitexin 2''-O-beta-D-glucoside | 1.2795** | 1.2358* | 1.0354 | sheath |
