# Supplementary figures and images for: UPLC-QTOF-MS metabolomics analysis revealed the contributions of metabolites to the pathogenesis of Rhizoctonia solani strain AG-1-IA
Source: PLoS One. 2018 Feb 6;13(2):e0192486. doi: 10.1371/journal.pone.0192486 (PMC5800620; doi:10.1371/journal.pone.0192486)

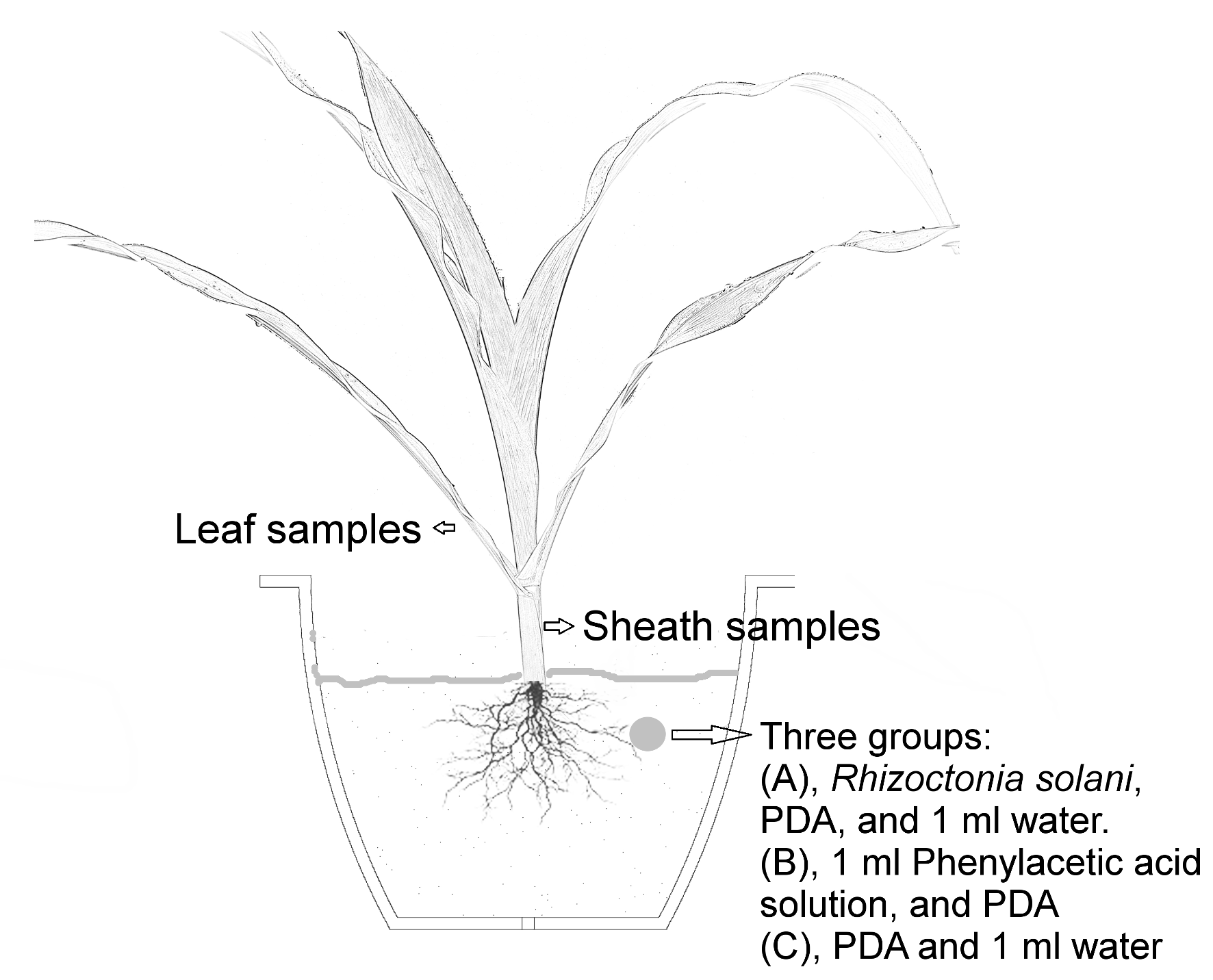

Supplement: S1 Fig — The inoculation of group A consisted of three PDA plugs with R. solani colonies and 1 ml distilled water. The inoculation of group B consisted of three PDA plugs and 1 ml phenylacetic acid solution. The inoculation of group C consisted of three PDA plugs and 1 ml distilled water. The leaf tissues that were close to the ground and 1 cm away from the sheath node were collected. The sheath tissues that were 5 cm away from the ground were sampled. (TIF) [file pone.0192486.s007.tif]

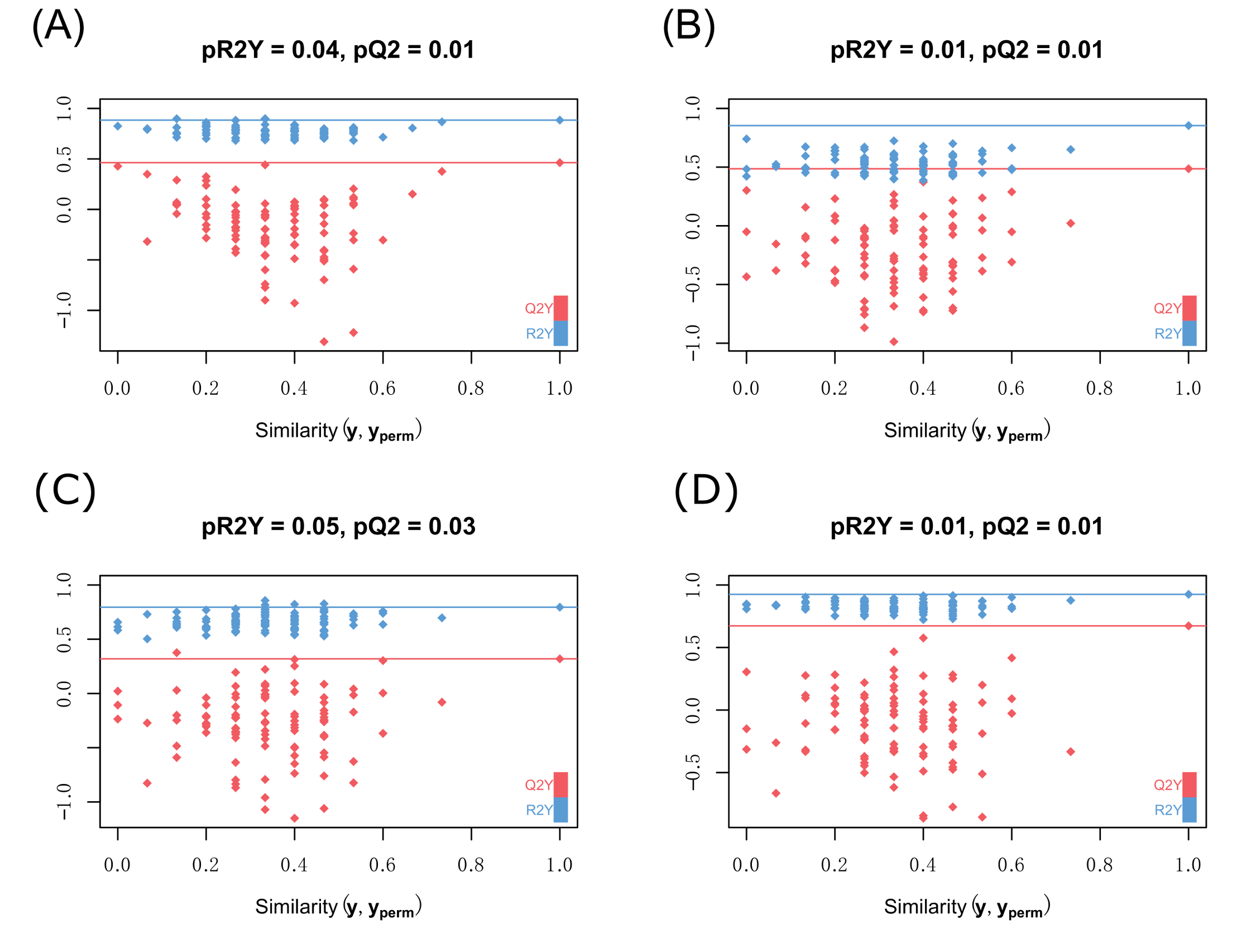

Supplement: S2 Fig — (A) and (B), the plots from the permutation test results of the PLS-DA models for the leaf tissues analyzed in positive and negative ion modes, respectively. (C) and (D), the plots from the permutation test results of the PLS-DA models for the sheath tissues analyzed in positive and negative ion modes, respectively. The number of each permutation test equals 100. The results suggested that all PLS-DA models were not over-fitted. (TIF) [file pone.0192486.s008.tif]

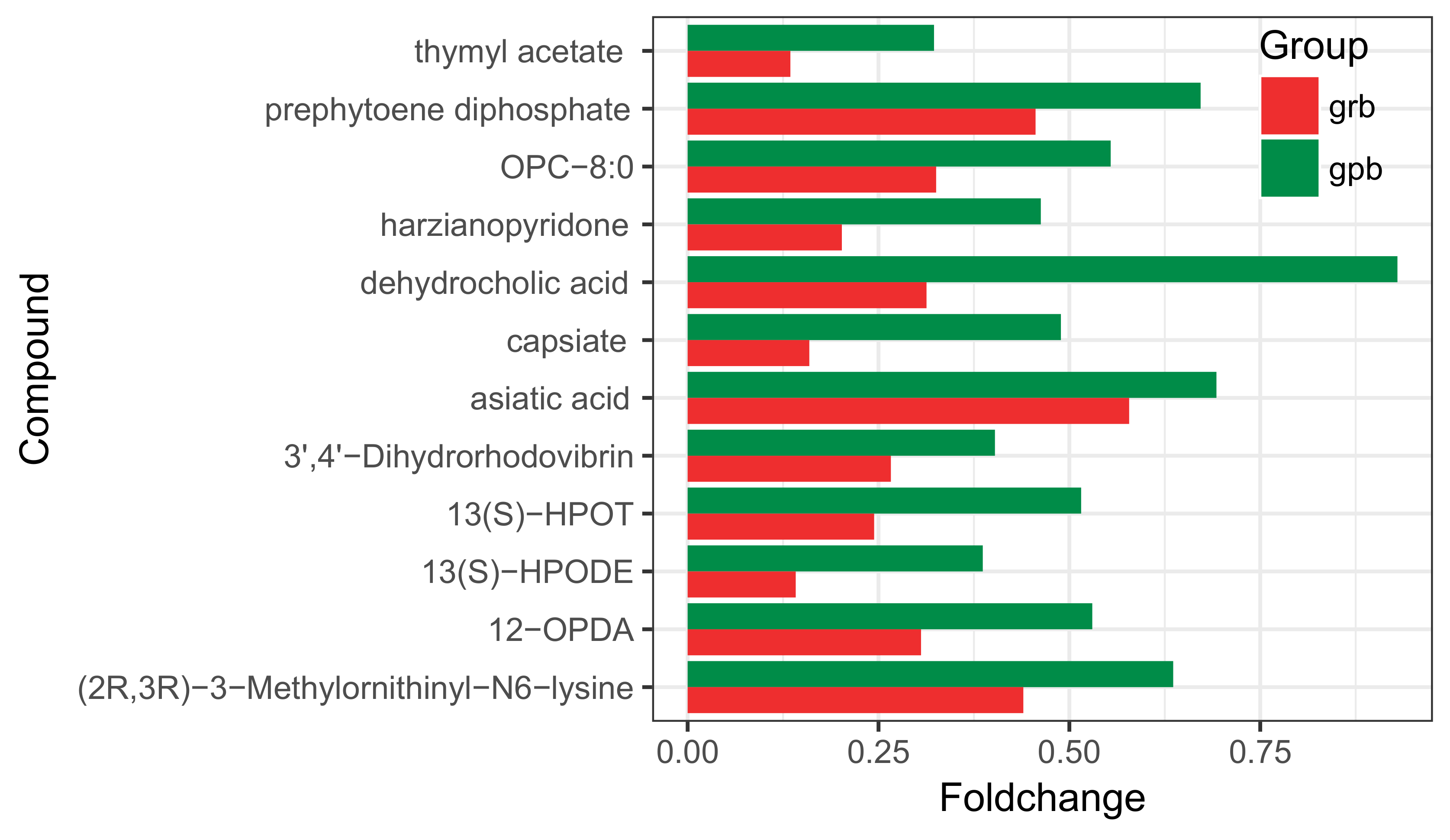

Supplement: S3 Fig — Grb and Gpb: Each value represented the ratio of the average concentration of a given metabolite in the R. solani (Grb) or phenylacetic acid (Gpb) treated leaf group divided by the average concentration of the same metabolite in the control leaf group. (TIF) [file pone.0192486.s009.tif]
